# Cutaneous Metastasis of Lung Adenocarcinoma: Initially Mimicking and Misdiagnosed as Keloids

**DOI:** 10.7759/cureus.27285

**Published:** 2022-07-26

**Authors:** Jack B Newcomer, Abigail Durbin, Chase Wilson

**Affiliations:** 1 Dermatology, University of Kentucky College of Medicine, Lexington, USA; 2 Dermatology, Elkhorn Dermatology, Georgetown, USA

**Keywords:** cutaneous metastasis, metastatic adenocarcinoma of lung, dermatology case report, dermatology consultation, cutaneous oncology, clinical dermatology, cutaneous malignancy

## Abstract

Cutaneous metastases have distinct morphologic features that can aid in making the diagnosis clinically even prior to biopsy. Lesions often have a nodular appearance and are firm, fixed, and range from flesh-colored to reddish-purple. A 73-year-old female with a history of lung adenocarcinoma status-post neoadjuvant chemotherapy and lobectomy 20 months prior was referred to our dermatology clinic for evaluation and treatment of suspected keloids on the left flank. The lesions were firm, plum-colored, fibrotic nodules, and were diagnosed clinically in the office as cutaneous metastases of internal malignancy. Punch biopsy was performed and revealed a proliferation of atypical epithelial cells arranged in cords and strands, with neoplastic cells positive for CK7 and TTF-1, confirming the diagnosis of metastatic adenocarcinoma. The patient was referred for chemotherapy and is still alive nine months following the prompt clinical diagnosis of cutaneous metastasis. Cutaneous metastasis signifies a poor prognosis, but knowledge of the clinical characteristics of these lesions can lead to earlier detection and more prompt initiation of treatment.

## Introduction

The skin is an uncommon site of metastasis from internal malignancies, with cutaneous metastases encountered in 1-10% of all patients with cancer [1]. In most cases, cutaneous metastases are identified after the initial diagnosis of the primary malignancy. However, in some cases, especially with malignancies that remain clinically silent for long periods of time, cutaneous metastasis can be the initial sign that leads to the diagnosis of an internal malignancy [2]. 

in the setting of lung cancer, the most frequent sites of metastasis are the brain, bone, liver, and adrenal glands. Skin metastases occur much less frequently, with one meta-analysis showing an incidence of 3.4% in patients with lung cancer. Cutaneous metastases in the setting of lung cancer most commonly present as firm, painless, nodules on the chest, abdomen, or head and neck region, but can present in a variety of forms and mimic benign lesions [3]. Skin metastases from primary lung cancer confer a poor prognosis, especially given they usually present with other internal metastases [4-5]. Cutaneous metastasis should be considered for any suspicious lesion in patients with a history of lung cancer or smoking.

We present a case of a 73-year-old female with a history of adenocarcinoma of the lung who was initially referred for evaluation and treatment of keloids but was diagnosed with metastatic adenocarcinoma. The aim of this case is to promote awareness of the morphology of skin metastasis so that physicians can diagnose these lesions clinically with minimal delay in diagnosis and treatment.

## Case presentation

**Figure 1:**
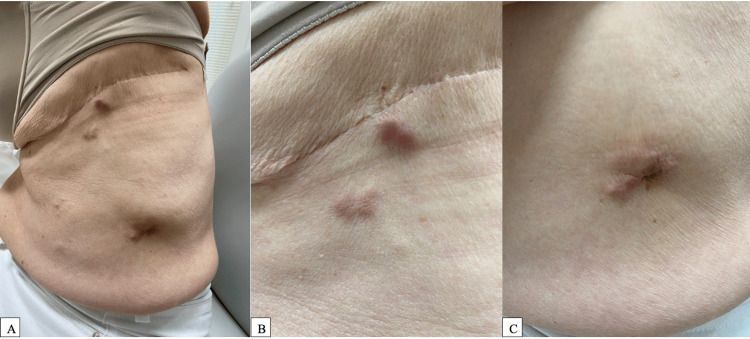
Three fixed, fibrotic, plum-colored nodules on the left abdomen and flank, consistent with cutaneous metastasis of underlying adenocarcinoma of the lung. Full image of the entire affected area (1A), as well as close-up views of the more superior lesions (1B) and inferior lesion (1C).

A 73-year-old female with a history of lung adenocarcinoma status-post neoadjuvant chemotherapy (three cycles of cisplatin/nivolumab/pemetrexed) and left upper lobectomy 20 months prior with adjuvant radiation was referred by her primary care physician for evaluation of suspected keloids on the left side of her abdomen (Figures 1A-1C). The lesions were red, itchy, and purulent, and had been present for three months. On physical exam, there were three distinct plum-colored, fixed, firm, fibrotic nodules located on the left flank adjacent to the patient’s prior lobectomy scars. The differential diagnosis at the time included cutaneous metastases from recurrence of the patient’s primary lung cancer, keloids, lymphoma cutis, and atypical fungal or mycobacterial infection.

A 4-mm punch biopsy of the lesion was performed given the suspicion for malignancy. Biopsy results revealed ulceration of the surface with an inflammatory crust, along with a proliferation of atypical epithelial cells present diffusely between and among collagen bundles of the dermis. The neoplastic cells were arranged in cords and strands and positive for CK7 and TTF-1, and negative for CK20. This pattern is suggestive of metastatic adenocarcinoma and was diagnosed as so in the pathologic report, compatible with the primary lung cancer. 

**Figure 2:**
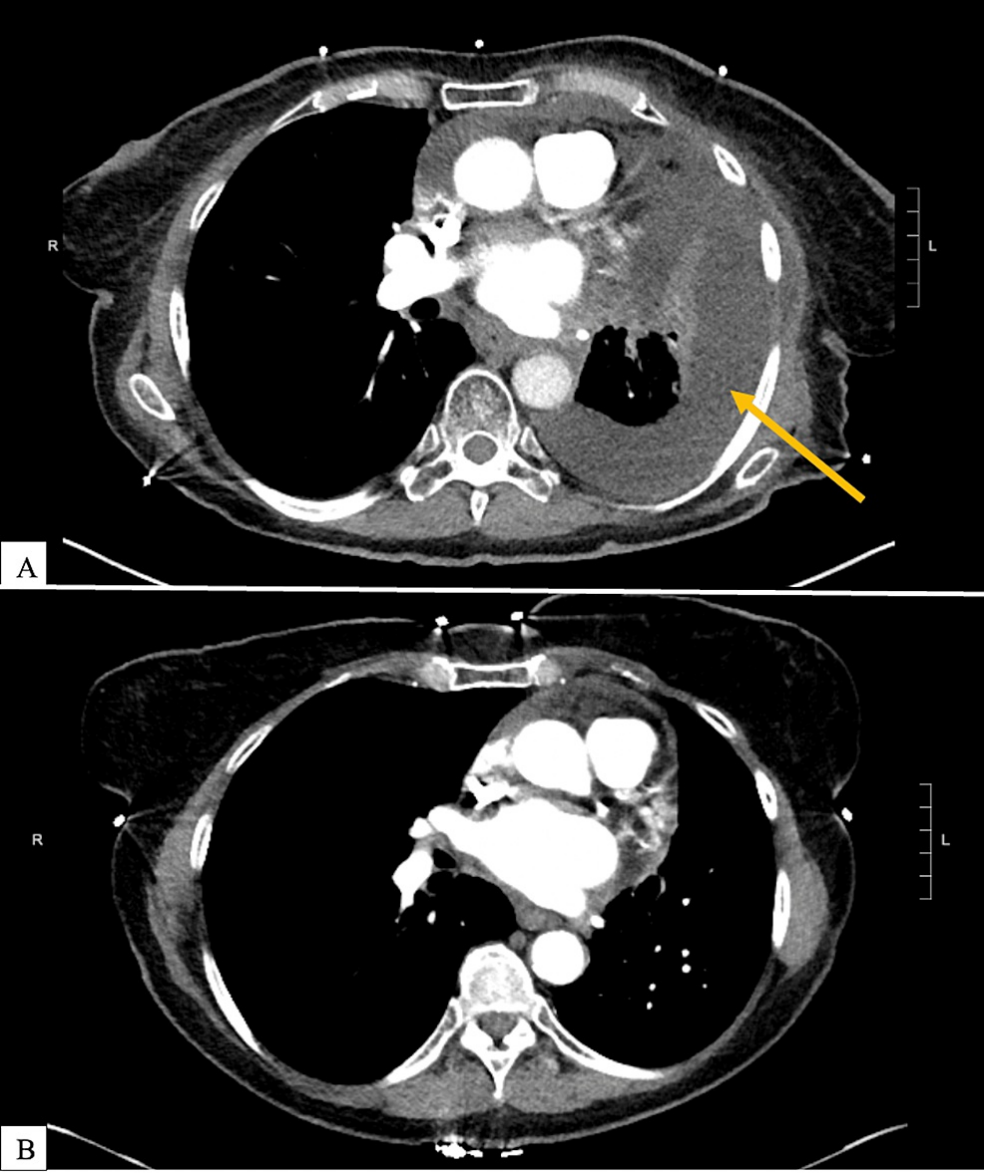
CT pulmonary angiogram at the time of hospital presentation (a) revealing a large loculated left pleural effusion (arrow), with near complete collapse of the left lower lobe. A CT chest from approximately the same level from seven months prior (b) showing only minimal pericardial effusion and absence of pleural effusion or atelectasis.

Two days following the presentation to our clinic, the patient presented to an academic medical center with worsening shortness of breath. CT scan revealed a large loculated left pleural effusion, significantly increased in size compared to imaging seven months prior (Figures 2A-2B). A video-assisted thoracoscopic surgery (VATS) was performed with a biopsy taken of one of many visible pleural nodules. Biopsy results revealed adenocarcinoma, compatible with the primary tumor. A chest tube was placed and left to drain the fluid collection. The patient began chemotherapy two weeks later and has since completed six of six planned cycles with carboplatin and pemetrexed, and is now taking oral palbociclib. She is still alive nine months following diagnosis of cutaneous metastasis with currently stable disease. 

## Discussion

The skin is an uncommon site of metastasis when compared with other organs. Mollet et al. showed in a systematic review of cutaneous metastasis that the most common sources of cutaneous metastases in men are the lung (24%), colon (19%), melanoma (13%), and oral cavity (12%). The most frequent malignancies that metastasize to the skin in women are breast (69%), colon (9%), melanoma (5%), ovarian (4%), and lung cancer (4%) [6]. In the setting of lung cancer, metastatic disease most commonly occurs in the brain, bone, liver, and adrenal glands, with skin metastasis occurring much less frequently. Metastasis to the skin is usually identified in later stages of the disease after diagnosis of the primary malignancy, but in some cases can be the first indication of malignancy, especially in primary tumors that remain clinically silent for relatively longer periods of time. 

The appearance of cutaneous metastases varies widely and can mimic benign skin lesions, particularly when they are a single lesion [7]. The most common appearance of skin metastasis is a firm, painless, nodule, but metastatic lesions can also develop as papules, plaques, ulcers, bullae, cellulitis-like lesions, or fibrotic lesions. Nodules are usually fixed, firm, and painless. They are often flesh-colored but can vary in color from red-purple to blue-black, and vary in diameter from 2 mm to 6 cm. Cutaneous metastases can appear as single lesions or non-specific groups of discrete nodules that enlarge rapidly without any explanation [8]. Nodular metastatic lesions are often misdiagnosed as simple cysts or benign connective tissue lesions such as keloids [9]. High clinical suspicion of a primary or recurrent neoplasm can aid in the differential diagnoses.

In our patient, the lesions were initially diagnosed as keloids prior to referral to our clinic. Although keloids can also present as firm, pink, nodules and be similar in size to cutaneous metastases, keloids are softer and directly overlay surgical scars, skin piercings, or other areas of skin trauma. The lesions present on our patient were adjacent to her prior surgical scar, rather than directly overlying it. One study showed that 86% of patients with keloids complained of pruritus, while 46% experienced pain [10]. Keloids are also typically associated with preceding skin trauma or inflammation, and the lesions often extend beyond the margins of the initial wound or injury. Although our patient’s lesions shared some similar clinical characteristics to keloids, the lesions were suspicious for metastasis given the fixed, painless nature of the lesions, the appearance of secondary ulceration, as well as the prior history of lung cancer. 

The anatomical distribution of the cutaneous metastasis often correlates with the origin of primary malignancy and the mechanism of metastatic spread. Internal malignancies generally metastasize to sites close to the primary tumor but are capable of metastasizing anywhere on the cutaneous surface. Lung, melanoma, and breast cancers are the most common malignancies to metastasize to remote cutaneous sites [11]. The most common sites for cutaneous metastases from lung cancer in men are the chest and the head and neck region. In women, the anterior chest wall and the abdomen are the most commonly involved sites. These findings are more relevant in cases where skin metastasis is the presenting sign of malignancy. In cases with a known history of malignancy, any suspicious lesion should prompt biopsy for evaluation of metastasis from the primary tumor. 

Immunohistochemical markers can assist in the identification of the primary cancer, as cutaneous metastases from internal malignancies are indistinguishable from one another by their appearance on a physical exam alone. Expression of CK7 is positive in cancers of the lung, breast, ovary, pancreas, thyroid, and salivary gland, whereas expression of TTF-1 is characteristic of primary lung cancer and thyroid cancer. When a thyroid primary is ruled out, TTF-1 expression is both sensitive and specific for primary adenocarcinomas, bronchioloalveolar carcinomas, and small-cell carcinomas [12]. Expression of CK20 is positive in cancers of the colon, stomach, and Merkel cells, and is negative in cancers of the lung. Positivity with stains such as cytokeratin ⅚ and p63 supports primary cutaneous origin over metastasis. The positive staining of CK7 and TTF-1 combined with negative CK20 in our patient was highly suggestive of primary adenocarcinoma of the lung. 

Cutaneous metastases are a sign of poor prognosis. A retrospective analysis of patients with biopsy-proven skin metastases performed by Sariya et al. showed that 86% (43/50) of patients with cutaneous metastases had known stage IV cancer, and the mean interval between diagnosis of the primary malignancy and the development of skin metastasis was 36 months (ranging from 1-177 months) [7]. Skin metastasis was the presenting sign in six (14%) of these patients, and the internal malignancy was discovered simultaneously in one of these patients and within one month in the other five. In patients with primary lung carcinoma, the average survival following the identification of metastatic skin lesions ranges from three to five months [13-15]. Therefore, only palliative chemotherapy is traditionally offered following the discovery of cutaneous metastasis. The patient in our case is still alive at nine months post-detection of metastasis and is tolerating chemotherapy well with stability of her underlying disease.

## Conclusions

Cutaneous metastases from the lung are relatively uncommon and are a sign of poor prognosis. They most commonly present as nodular lesions on the chest, abdomen, or head and neck region, but can present in a variety of other forms and on any cutaneous surface. Although metastases can mimic benign lesions such as keloids, there are distinct clinical differences that can help clinicians to diagnose metastases early and limit delays in initiation of treatment. Lesions that are painless, firm, fixed, or associated with secondary ulceration, especially in combination with a history of internal malignancy should raise suspicion for cutaneous metastasis. In patients with a history of smoking or lung cancer, any suspicious skin lesions should prompt full thickness skin biopsy using a punch tool or incisional biopsy for histological diagnosis of possible metastasis. 
